# Early-Life Nutritional Determinants of Pediatric MASLD

**DOI:** 10.3390/nu17243871

**Published:** 2025-12-11

**Authors:** Johanna K. DiStefano

**Affiliations:** Translational Genomics Research Institute, Phoenix, AZ 85004, USA; jdistefano@tgen.org; Tel.: +602-343-8814

**Keywords:** pediatric MASLD, developmental programming, maternal obesity, gestational diabetes, breastfeeding, infant formula, added sugars, early-life nutrition, hepatic steatosis, metabolic imprinting

## Abstract

Metabolic dysfunction-associated steatotic liver disease (MASLD) is the most prevalent chronic liver disorder in both children and adults. Pediatric MASLD, however, is not simply an early form of adult disease, as it exhibits distinct developmental, histological, and metabolic features. Emerging evidence suggests that these characteristics arise from a complex, multi-hit continuum that begins in utero. Maternal obesity, gestational diabetes, and poor diet quality during pregnancy have been associated with greater hepatic steatosis in offspring, raising the possibility that intrauterine exposure to dyslipidemia, hyperglycemia, and elevated free fatty acid flux may contribute to early hepatic lipid deposition. After birth, feeding behaviors such as a prolonged breastfeeding appear protective, whereas formula feeding, especially high added-sugar formulations, may accelerate rapid weight gain and increase susceptibility to later steatosis. Early childhood diets high in added sugars, saturated fats, and ultra-processed foods may further promote hepatic lipogenesis and inflammation and interact with underlying genetic susceptibility. Given the heterogeneity of available human cohort studies and mechanistic model systems, this narrative review summarizes converging evidence from prenatal, postnatal, and early childhood nutritional exposures and their relationship to offspring hepatic lipid accumulation, emphasizing early-life windows for intervention to reduce the burden of pediatric MASLD.

## 1. Introduction

Over the past two decades, the landscape of pediatric chronic disease has been profoundly shaped by metabolic dysfunction-associated steatotic liver disease (MASLD), now recognized as the most common chronic liver condition among children worldwide [1,2]. MASLD encompasses a spectrum of progressive liver disorders defined by excessive hepatic fat accumulation in the context of metabolic dysfunction. In children, MASLD is diagnosed when hepatic steatosis is detected by imaging or histology in the presence of at least one cardiometabolic risk factor, such as obesity, dyslipidemia, dysglycemia, or hypertension [3]. While many children exhibit simple hepatic steatosis (defined by ≥5% liver fat content), a clinically meaningful subset progress to metabolic dysfunction-associated steatohepatitis (MASH), a more severe condition characterized by steatosis, hepatocellular injury (e.g., ballooning degeneration), and lobular inflammation, often accompanied by fibrosis [3,4]. MASLD serves as an umbrella term comprising the full spectrum of disease from hepatic steatosis to MASH.

The development of inflammation and fibrosis in adult MASLD typically unfolds over decades [5]. In contrast, MASH can emerge in children and adolescents within only a few years of the onset of steatosis or liver injury [6,7,8]. This accelerated, more aggressive trajectory suggests that pediatric disease is not simply an early manifestation of adult MASLD but instead reflects a distinct clinical and biological entity. Features such as portal-predominant inflammation and periportal fibrosis [9], as opposed to the lobular inflammation typical of adults, are consistent with age-specific mechanisms of injury and repair and may partially explain why MASLD confers a higher mortality risk in young adulthood [10,11,12,13]. These histological differences underscore the fundamental divergences in pediatric and adult disease pathogenesis.

More specifically, pediatric disease often presents with portal-based (zone 1) inflammation and fibrosis, whereas adult disease typically shows pericentral (zone 3) injury and perisinusoidal fibrosis [9,14,15]. Schwimmer et al. [15] delineated two histologic sub-types of pediatric steatohepatitis, one characterized by ballooning degeneration with zone 3 fibrosis and another defined by zone 1 portal inflammation and fibrosis, the latter associated with younger age and more rapid progression of fibrosis. Additionally, zone 1 involvement correlates with advanced fibrosis and earlier disease onset, while zone 3 predominance has been linked to steatohepatitis [14]. These zonal patterns may reflect developmental differences in hepatic metabolism, oxygen and nutrient gradients, and immune-parenchymal signaling [9].

The public health implications of pediatric MASLD are considerable. In the United States, MASLD affects a substantial portion of youth, with prevalence estimates ranging from 5–20% among children and adolescents [16,17,18,19]. This burden is tightly connected to the obesity epidemic: approximately 20% of U.S. adolescents (ages 12–17) meet the criteria for obesity, and of these, ~70% have MASLD [20]. Globally, the disease burden is increasing and regionally variable. Overall prevalence approaches 13% [1], and rates have risen by 26.7% between 1990 and 2019 [21]. Regional disparities are pronounced, with prevalence in North America as high as ~40% and lower estimates in Africa (~30%) [1]. Obese youth have particularly high prevalence (up to 47%) [2], yet a significant proportion of pediatric MASLD occurs in children with a normal weight [22,23], mirroring patterns observed in adults [24]. Because most children are asymptomatic and diagnosis is often incidental, frequently discovered via elevated alanine aminotransferase (ALT) levels or imaging performed during obesity screening, current prevalence estimates likely underestimate the true burden [25].

Pediatric MASLD is multifactorial, arising from the interplay of genetic predisposition, developmental programming, and environmental exposures. The Developmental Origins of Health and Disease (DOHaD) theory posits that early nutritional and metabolic exposures establish long-term physiological set points, influencing later disease susceptibility [26,27]. Pediatric MASLD exemplifies this concept, as intrauterine and early postnatal nutritional exposures can program hepatic lipid metabolism, insulin sensitivity, and inflammatory tone, shaping disease trajectory from early life.

A strong heritable component is supported by familial clustering [28,29], as well as associations of *PNPLA3*, *TM6SF2*, and *GCKR* variants—known to confer susceptibility in adults—with hepatic steatosis and fibrosis progression in children [30,31,32,33]. These common variants influence hepatocellular fat storage, lipid export, and glucose-lipid partitioning [34]. Notably, the *PNPLA3* I148M allele appears to enhance sensitivity to high sugar intake and rapid postnatal weight gain, highlighting gene-nutrition interactions in early-life programming [35,36]. Ethnic and racial background also influences disease risk, with higher prevalence in Hispanic and Asian youth [20,29].

Although genetic and epigenetic factors contribute to inter-individual risk, they are beyond the primary focus of this review. Early nutritional exposures can influence epigenetic marks, including DNA methylation and microRNA patterns, affecting pathways of lipid metabolism, and have been reviewed elsewhere [34,37,38,39]. Early-life factors, including maternal obesity, insulin resistance, gestational diabetes, and abnormal birth weight further modulate susceptibility [9], in alignment with the DOHaD model. These differences persist even after accounting for obesity and likely reflect a combination of genetic, social, and metabolic determinants, including dietary patterns and adipose tissue distribution [9].

As in adult disease, environmental and lifestyle factors are key drivers and important targets for preventing and managing pediatric MASLD. For example, poor diet quality [40,41,42,43,44,45,46,47] and low physical activity levels, both common in children with MASLD, contribute to hepatic fat accumulation and metabolic dysfunction [45,48,49]. These modifiable factors provide opportunities for early, non-pharmacologic intervention. Given the potential influence of early-life nutritional exposures [50], the purpose of this brief narrative review is to synthesize evidence linking prenatal, postnatal, and early childhood nutrition to the developmental programming of hepatic lipid accumulation.

To narrow the scope, this review focuses on nutritional and metabolic determinants across the full spectrum of pediatric MASLD and does not explore mechanistic domains such as epigenetic regulation, paternal contributions, or microbiome-mediated effects, for which readers are referred to recent comprehensive reviews [9,51,52]. For consistency with current nomenclature, the term MASLD is used throughout this review. However, most of the cited literature predates the 2023 consensus reclassification [3], using the terms nonalcoholic fatty liver disease (NAFLD) or nonalcoholic steatohepatitis (NASH). While the terminology has changed, the underlying phenotype of excess hepatic fat accumulation in the context of metabolic dysfunction remains largely comparable.

## 2. Methods

This narrative review was conducted through comprehensive searches of the PubMed and Scopus databases, encompassing literature published through October 2025. Searches were restricted to English-language articles. The search strategy utilized combinations of keywords related to maternal diet, early childhood nutrition, and pediatric MASLD. Search terms included combinations of “maternal obesity”, “maternal diet”, “gestational diabetes”, “fetal programming”, “breastfeeding”, “infant formula”, “added sugars”, “postnatal diet”, “pediatric nutrition”, “hepatic steatosis”, “liver fat”, “liver fibrosis”, “NAFLD”, “NASH”, “MASLD”, “MASH”, and “MAFLD”.

Longitudinal cohort studies, randomized controlled trials, and recent systematic reviews that examined maternal or early-life nutritional exposures in relation to hepatic steatosis or MASLD-related outcomes were prioritized. Human studies were weighed most heavily, given the focus on pediatric disease trajectories, while mechanistic animal and translational studies were selectively included when they provided biological insights that complemented human evidence. Additional relevant studies were identified through manual screening of reference lists of included articles.

For each eligible human study, information on study design, sample size, participant characteristics, exposure definitions, hepatic outcomes, and major findings was extracted to support consistent interpretation across the evidence base.

## 3. A Brief Overview of Pediatric MASLD Pathophysiology

MASLD is ultimately a systemic disorder of energy metabolism [53]. Insulin resistance in adipose tissue and muscle elevates circulating fatty acids and alters adipokine profiles, while gut dysbiosis increases intestinal permeability and exposure to gut-derived endotoxins [54]. These extrahepatic disturbances create a feed-forward loop of lipotoxicity, inflammation, and fibrogenesis [55].

**Figure 1:**
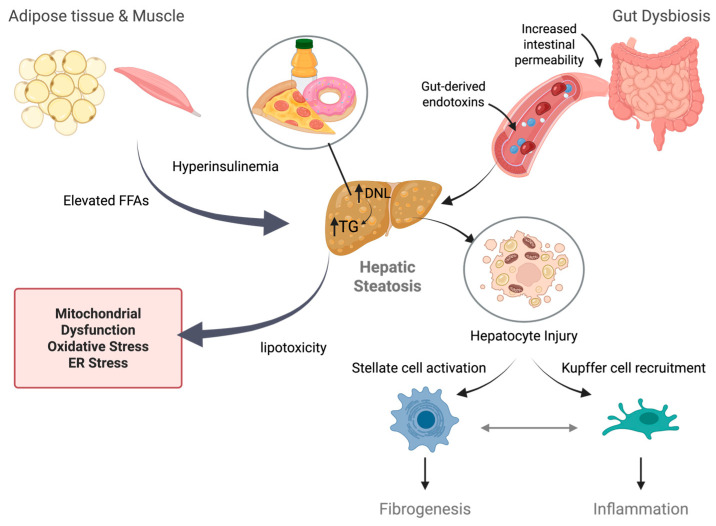
**Key features of MASLD pathophysiology**. See text for details. The figure was created by the author using Biorender (https://www.biorender.com/, accessed on 1 December 2025).

Early-stage MASLD is characterized by hepatic steatosis, which arises from an imbalance between lipid accretion and disposal. Excess delivery of free fatty acids from adipose tissue, enhanced de novo lipogenesis driven by both hyperinsulinemia and high dietary fructose intake, and impaired β-oxidation collectively promote triglyceride accumulation in hepatocytes [56]. When lipid storage capacity in hepatocytes is exceeded, metabolic pathways become dysregulated, leading to the generation of toxic lipid intermediates such as ceramides, diacylglycerols, and free cholesterol, and inducing mitochondrial dysfunction, oxidative stress, and endoplasmic reticulum stress (Figure 1).

These processes activate the unfolded protein response, generate reactive oxygen species, and trigger hepatocyte injury accompanied by the release of damage-associated molecular patterns (DAMPs) [57,58,59,60]. DAMPs are then recognized by pattern recognition receptors on immune cells, which triggers inflammatory cascades [61]. This process stimulates hepatocytes to release cytokines and chemokines that subsequently recruit Kupffer cells and infiltrating macrophages. Concurrently, mitochondrial DNA and lipid peroxidation products activate innate immune pathways such as TLR4 (toll-like receptor 4) and NLRP3 (NOD-, LRR- and pyrin domain-containing protein 3) inflammasome signaling [62]. Chronic inflammation further disrupts insulin signaling, perpetuating a cycle of metabolic stress and cellular injury. Persistent hepatocyte stress also activates hepatic stellate cells, promoting extracellular matrix deposition and fibrogenesis [60].

## 4. Maternal Nutrition and Offspring Hepatic Steatosis

Observational studies support associations between maternal obesity, gestational diabetes mellitus (GDM), and increased hepatic fat in offspring [9,52] (Table 1). Using magnetic resonance spectroscopy (MRS), Modi et al. [63] observed an 8.6% increase in neonatal intrahepatic lipid per unit increase in maternal BMI, independent of neonatal body weight. Similarly, Brumbaugh et al. [64] reported 68% higher hepatic fat in neonates born to obese mothers with GDM compared to those of normal-weight women, with intrahepatic lipid content correlating with maternal BMI rather than neonatal adiposity. These findings were among the first to suggest that fetal hepatic steatosis can occur independently of generalized fat accretion and imply that lipid partitioning to the fetal liver is affected by adipose storage-independent pathways.

In the longitudinal Western Australia Pregnancy (Raine) Cohort study, maternal pre-pregnancy obesity predicted MASLD at 17 years of age, independent of a Western-style diet consumption [65]. Likewise, in the Shanghai Prenatal Cohort (SPCS), maternal obesity and GDM were independently associated with hepatic steatosis in 8-year-old offspring [66]. The prevalence of hepatic steatosis rose across maternal BMI quartiles, and children of women with both obesity and GDM exhibited an eightfold-greater odds of steatotic liver compared to those of normal weight, normoglycemic mothers.

Comparable associations have been documented in large longitudinal cohorts with extended follow-up, including the Avon Longitudinal Study of Parents and Children (ALSPAC) and the ESPRESSO (Epidemiology Strengthened by Histopathology Reports in Sweden) studies. The ALSPAC cohort, with follow-up into young adulthood, demonstrated a persistent association between maternal adiposity and offspring hepatic fat [67,68]. Maternal pre-pregnancy overweight and obesity conferred higher risk of hepatic steatosis, whether assessed by ultrasound in adolescence (17–18 years) [68] or transient elastography in young adulthood (24 years) [67]. At both time points, this association was largely mediated by the offspring’s concurrent adiposity, but not by birthweight or breastfeeding, suggesting that the principal mechanism involves interplay between intrauterine metabolic programming and postnatal lifestyle trajectories. In contrast, the influence of maternal diabetes or glycosuria appeared to diminish over time: it was a strong, independent predictor of adolescent hepatic steatosis [aOR 6.74 (95% CI 2.47, 18.40)] [68], but was substantially attenuated and nonsignificant by early adulthood [OR 1.39 (95% CI 0.87, 2.21)] [67]. Excess gestational weight also increased steatosis risk, though this effect was primarily mediated by offspring BMI. In the ESPRESSO study, a population-based case–control design leveraging national registry data, maternal obesity was associated with biopsy-proven MASLD and greater disease severity in adult offspring (≤25 years of age), independent of GDM, smoking, and socioeconomic factors [69].

**Table 1:** Observational studies of maternal BMI or diet quality and offspring hepatic fat.

Cohort	Design	Loc	*n*	Age	Modality	Main Findings
Children’s Hospital CO [64]	CC	USA	25	1–3 wks	MRS	Greater IHCL content in neonates born to obese diabetic mothers
Chelsea & Westminster Hospital [63]	CS	UK	105	11.7 d	MRS	8.6% IHCL increase per BMI unit
Raine [65]	L	Aus	1170	17 y	USS	Maternal obesity increases adolescent MASLD risk, breastfeeding >6 months confers protection
ALSPAC [68]	L	UK	1215	17–18 y	USS	Offspring adiposity mediates maternal obesity/diabetes steatosis risk
ALSPAC [67]	L	UK	3353	24 y	TE	
ESPRESSO [69]	CC	Sweden	165	<25 y	Biopsy	Maternal obesity increases MASLD/severe MASLD in young adults
SPCS [66]	L	China	430	8 y	TE	Offspring steatosis aOR 8.26 for maternal obesity and GDM
Healthy Start [70]	L	USA	278	4–8 y	MRI	Poor maternal diet increases offspring steatosis susceptibility

CC: case–control; CS: cross-sectional; L: longitudinal; Loc: location; MRS: magnetic resonance spectroscopy; USS: abdominal ultrasound; TE: transient elastography; MRI: magnetic resonance imaging; CO: Colorado.

In addition to obesity and GDM, maternal MASLD is emerging as a determinant of offspring metabolic liver risk [71]. Though this area remains underexplored, studies on maternal hepatic steatosis and its associated metabolic dysfunction have been linked to higher adiposity, altered lipid partitioning, and early markers of hepatic fat accumulation in offspring [72,73,74].

### 4.1. Connecting Human Observational Findings and Mechanisms

Human studies indicate that both maternal metabolic status and diet quality during pregnancy independently modulate offspring risk of hepatic steatosis. However, heterogeneity exists in the timing and modality of hepatic outcome assessment, ranging from infancy to adulthood and from ultrasound to biopsy. Mediation analyses suggest that while postnatal adiposity accounts for part of the association, the persistence of prenatal effects even after adjustment supports the idea that intrauterine exposure may establish a “metabolic memory” within the developing liver. The consistency of findings across ethnically diverse cohorts supports an independent effect of maternal metabolic dysregulation on offspring hepatic lipid handling. Rare histologic evidence from autopsy studies of stillborn infants reinforces this link by eliminating postnatal confounding. In a retrospective autopsy study, Patel et al. [75] found macrovesicular steatosis in 79% of stillborn infants (*n* = 33) of diabetic mothers, compared to 17% of infants (*n* = 48) born to normoglycemic mothers. The severity of steatosis correlated with gestational age and fetal weight. Although this study was limited by the grouping of all maternal diabetes types (type 1, type 2, and gestational) into a single category and potential postmortem lipid redistribution, its findings suggest that maternal hyperglycemia and dyslipidemia alone can induce substantial fetal hepatic lipid deposition, independently of shared postnatal factors [75].

**Figure 2:**
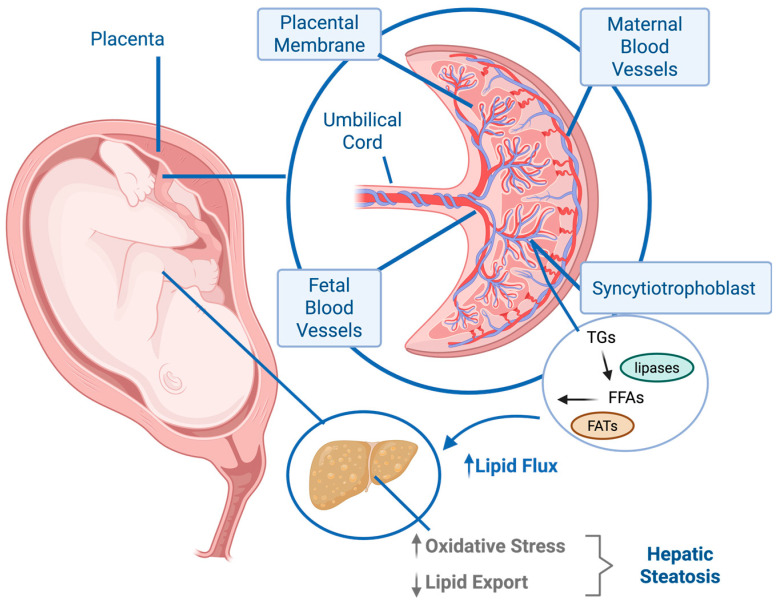
**Mechanisms of fetal lipid overload in maternal obesity.** Elevated maternal triglycerides (TGs) and free fatty acids (FFAs) characteristic of excess adiposity and gestational diabetes are delivered to the placenta. Placental lipases hydrolyze TGs, generating FFAs that are transported across the syncytiotrophoblast via fatty acid transporters (FATs), such as FATP, FABP, and CD36. The resulting increase in lipid flux to the fetus overwhelms hepatic oxidative and VLDL export capacities, leading to intrahepatic lipid accumulation and fetal hepatic steatosis. The figure was created by the author using Biorender. ↑: increase; ↓: decrease.

During normal pregnancy, maternal triglycerides and free fatty acids rise to support fetal growth. However, mothers with obesity and gestational diabetes experience a sharper increase, with triglyceride levels ~ 40–50% higher than those of normal-weight women throughout early to late gestation [76]. This maternal hypertriglyceridemia, influenced by both maternal body composition and dietary fat consumption, modulates placental lipid processing and contributes significantly to offspring metabolic health [77]. Barbour and Hernandez [76] termed the resulting process “making fat from fat” whereby elevated maternal triglycerides, hydrolyzed by placental lipases, are transferred as free fatty acids to the developing fetus (Figure 2). Because the fetal liver has limited oxidative capacity and inefficient VLDL transport, it is poorly equipped to manage this significant lipid flux. Ultimately, this excess lipid accretion drives increased fetal adiposity, a key predictor of childhood obesity and lifelong metabolic dysfunction [78,79].

### 4.2. Insights from Animal Models

The placenta plays a critical intermediary role in the metabolic programming of the fetus. Maternal hyperglycemia, insulin resistance, and hypertriglyceridemia alter placental lipid storage, lipoprotein lipase activity, and fatty acid transport protein expression, thereby modifying the quantity and composition of lipids delivered to the fetus [80,81,82]. Placental inflammation, commonly observed in pregnancies complicated by obesity or GDM, further disrupts trophoblast mitochondrial function and nutrient-sensing pathways, amplifying the delivery of free fatty acids to the fetal circulation [83,84,85].

Understanding these complex, in utero mechanisms—and the fetal organ responses—is an ongoing area of investigation, necessitating the use of animal models to define placental transport [77]. However, species differences in placental architecture and placental lipid handling complicate extrapolation from animal models. For example, while rodent models have been vital for understanding the long-term metabolic programming in offspring, they frequently differ from the human phenotype, sometimes resulting in a fetal growth restricted phenotype rather than overgrowth [86,87,88,89,90].

Despite these caveats, non-human primate studies have yielded informative insights into maternal programming of offspring hepatic steatosis. Studies in macaques and baboons exposed to a high calorie, high fat, or high fructose diet observed a substantial increase in fetal hepatic triglycerides, oxidative stress, and upregulation of lipogenic and gluconeogenic genes during late gestation that often persisted after birth [91,92,93]. A Western-style (i.e., high-sugar, high-fat, high-calorie, low fiber) maternal diet also induced bile acid dysregulation, mitochondrial impairment, and altered immune programming in macaque offspring [94] and resulted in periportal collagen deposition and stellate cell activation in fetal macaque liver [95,96]. This pattern persisted in juvenile offspring even after weaning to a control diet [95]. However, modification of the obesogenic diet back to a control diet prior to a second pregnancy normalized fetal oxygenation and reduced markers of hepatic lipotoxicity and oxidative stress [96], indicating that maternal nutrition exerts lasting, diet-responsive effects on offspring hepatic physiology.

Recent studies in other animal models provide additional mechanistic context for maternal dietary influences on offspring hepatic metabolism. In rats, high-fat feeding during gestation and lactation induced hepatic steatosis in offspring, accompanied by increased body weight, impaired glucose tolerance, elevated serum cholesterol, and altered expression of lipogenic and beta-oxidation genes at weaning [97,98]. Similar effects were observed following maternal consumption of junk food or high-fat/high-sucrose diets, which promoted hepatic triglyceride accumulation, oxidative stress, insulin resistance, and downregulation of genes involved in lipid oxidation and VLDL transport [99,100]. When these diets were consumed during lactation, offspring exhibited greater total body fat and impaired liver function [101].

Excess maternal sugar intake and obesity exert additional diet-independent programming effects. Maternal fructose feeding, ranging from 10% to 63% of energy, induced maternal hypertriglyceridemia, fetal hyperinsulinemia, and altered leptin, SREBP-1c, and ACC2 signaling in rat offspring [102,103]. Perinatal obesity in mice primed a persistent hepatic metabolic stress response, characterized by impaired oxidative phosphorylation, dysregulated hepatokine expression, and long-lasting alterations in lipid metabolism, even in the absence of histological steatosis [104]. Postnatal exposure to a high-fat diet amplified these effects, exacerbating hepatic lipid accumulation and promoting ceramide deposition in offspring [105].

### 4.3. Macronutrient Composition and Hepatic Steatosis Risk

Overall, findings from human studies complement animal evidence. In the Healthy Start Study, poor maternal diet quality, characterized by high intakes of sugar and “empty calories”, and low consumption of green vegetables and legumes, was associated with greater hepatic fat in children aged 4–8 years [70]. Conversely, higher maternal fiber intake and adherence to a Mediterranean-style dietary pattern during pregnancy were linked to lower hepatic fat, independent of pre-pregnancy BMI [70]. Findings from the ALSPAC cohort showed a link between high free-sugar intake during pregnancy and hepatic steatosis in young adulthood [67]. Together, these studies suggest that maternal nutritional quality, in additional to maternal metabolic status, influences offspring hepatic lipid handling.

Gestational weight gain provides an integrated marker of overall maternal energy balance and diet quality [106,107]. Excess gestational weight gain, particularly when exceeding Institute of Medicine recommendations [108], has been associated with greater childhood adiposity and liver fat accretion [109,110,111]. Gestational weight gain has also been shown to partially mediate the relationship between maternal pre-pregnancy obesity and childhood adiposity [112]. Because gestational weight gain reflects cumulative dietary intake, macronutrient distribution, and maternal metabolic function, it likely captures broader aspects of the intrauterine nutritional environment that contribute to fetal hepatic lipid handling and susceptibility to later steatosis.

In addition to macronutrient composition, emerging studies indicate that maternal micronutrient status also influences fetal hepatic lipid metabolism. Choline, which is essential for phosphatidylcholine synthesis and VLDL export, contributes to hepatic fat partitioning [113]. Both human pregnancy cohorts and experimental models show that maternal choline insufficiency impairs fetal hepatic lipid packaging and promotes steatosis, particularly in the presence of maternal obesity or insulin resistance [114,115]. Similarly, low maternal vitamin D status has been associated with increased neonatal and childhood hepatic fat and may influence fetal hepatic insulin sensitivity [116,117]. Folate deficiency, through its role in one-carbon metabolism and epigenetic regulation, alters expression of genes involved in lipogenesis and β-oxidation [118,119,120]. Although these micronutrients have received less attention than macronutrient composition, available evidence supports their inclusion as nutritional determinants of early-life hepatic programming.

Emerging evidence further implicates maternal overnutrition in altering fetal immune and microbial programming. Fecal microbiota from infants of obese mothers induced hepatic inflammation, gut barrier dysfunction, and periportal injury in germ-free mice, recapitulating features of pediatric MASLD [121]. Related studies suggest that maternal insulin resistance and hypertriglyceridemia activate de novo lipogenic and inflammatory pathways in the fetal liver that persist despite post-weaning dietary normalization [122,123,124]. These findings support the hypothesis that the “first hit” in pediatric MASLD originates in utero through enduring metabolic and immunologic imprinting.

### 4.4. Modifiability of Early Life Programming: Evidence from Animal Models

Interventional animal studies provide crucial proof-of-principle that the programmed metabolic trajectory may be modifiable. For example, supplementation with the antioxidant pyrrolquinoline quinone during gestation and lactation in mice restored mitochondrial fatty acid oxidation, increased protective *n*-3 polyunsaturated fatty acids, and reduced triglyceride accumulation in offspring [125]. Furthermore, studies in non-human primates show the efficacy of dietary change: while maternal high-fat feeding decreased fetal *n*-3 fatty acids, increased the *n*-6:*n*-3 ratio, and promoted hepatic apoptosis, switching dams to a control diet prior to subsequent pregnancy normalized these adverse fetal outcomes [126]. Similarly, pre-conception diet reversal in obese macaques partially normalized fetal hepatic triglycerides, oxidative stress, lipogenic gene expression, and key metabolites, although effects like elevated ceramides persisted [96,127]. Targeted interventions, such as treating obese macaques on a Western-style diet with resveratrol, have also been found to reduce markers of hepatic injury including fetal hepatic collagen deposition, portal triad fibrosis, and oxidative stress, and fetal hypoxemia [127]. Collectively, these findings demonstrate that the adverse fetal hepatic programming induced by maternal overnutrition is not permanent and can be significantly mitigated through pre-conception or gestational nutritional and therapeutic interventions.

### 4.5. The DOHaD Framework

While the DOHaD hypothesis was initially framed around cardiovascular disease and type 2 diabetes [26,27], MASLD has now become accepted as an organ-specific extension of this concept. It is through the combination of intrauterine nutrient oversupply and subsequent postnatal dietary excess that the long-term hepatic lipid metabolism, insulin sensitivity, and inflammatory tone are programmed across the life course in offspring. Specifically, clinical, imaging, and experimental data confirm that maternal metabolic dysregulation can initiate hepatic lipid deposition and fibrogenic signaling in utero, thereby predisposing offspring to metabolic dysfunction later in life.

This developmental continuum, linking maternal status and early feeding to MASLD risk, is supported by a recent systematic review of maternal and perinatal factors influencing pediatric liver health [128]. Across studies, several risk and protective factors align with the DOHaD model (Table 2). While findings regarding gestational diabetes, preterm birth, and small-for-gestational-ages remain inconclusive or inconsistent, the collective evidence strengthens the developmental linkage between maternal metabolic status and early feeding patterns and subsequent pediatric MASLD risk.

## 5. Early Childhood Nutrition and Liver Fat Accretion

### 5.1. Postnatal and Infant Dietary Factors

The transition from exclusive milk feeding to complementary foods represents a sensitive window for shaping hepatic metabolism. Evidence from the Raine cohort indicates that breastfeeding without supplemental milk for ≥6 months reduces the odds of adolescent MASLD by 36%, independent of Western dietary pattern at 17 years of age [65]. Early introduction of formula or supplemental milk (<6 months) was associated with a higher prevalence and severity of steatosis and a more adverse metabolic profile. These findings are consistent with those of Nobili et al. [129], which showed a protective role for infant nutrition, with a longer duration of breastfeeding independently associated with a lower risk of biopsy-proven MASH and MASH fibrosis in later adolescence, even when accounting for age, waist circumference, gestational age, and birth weight (Table 3). However, an unrelated study showed that associations between infant feeding and general, visceral, and hepatic fat at age 10 were largely attenuated after adjustment for sociodemographic and maternal factors [130]. Examination at earlier postnatal timepoints (i.e., two months of age) detected no differences in adipose tissue or intrahepatocellular lipid accumulation between breastfed and formula-fed infants [131]. This observation may suggest that the substantial intrahepatocellular lipid increase seen early in life, independent of method of feeding, may be a normal physiological process or that the adverse effects of formula on liver fat are delayed beyond this early neonatal window.

In addition to feeding mode, the composition of human milk varies substantially among mothers and over the course of lactation [132,133,134]. Variations in human milk oligosaccharides, lipid species, inflammatory cytokines, and bioactive peptides influence gut maturation, insulin sensitivity, and early appetite regulation, all of which affect hepatic lipid handling [135,136,137,138,139]. It is possible that such differences in milk composition contribute to inter-individual variability in metabolic outcomes among breastfed infants.

Infant formula composition can further influence hepatic lipid accumulation. A recent analysis of commercial products showed that infant formulas contain significantly more medium-chain fatty acids compared with human milk [140]. Feeding high-energy, medium-chain fatty acid-rich formula to neonatal pigs induced hepatic steatosis and altered hepatocyte intermediary metabolism compared with isocaloric long-chain fatty acid formula [141]. Although extrapolation to human infants is limited, these findings indicate that the quality and chain length of dietary fats can influence hepatic lipid storage during early development.

Early sugar exposure may also be detrimental. In a population-based cohort of nearly 2000 infants, those consuming > 2 servings/day of sugar-containing beverages had a ~3-fold higher odds of MASLD at 10 years compared to infants consuming < 1 serving/day, with strongest effects among children of mothers with lower education levels and those who developed excess adiposity [142]. Although added sugar is not recommended for children < 2 years of age [143], most infant formulas sold in the United States primarily contain added sugars rather than naturally occurring lactose [144]. In an analysis of 73 commercial formulas, the median proportion of added sugars was ~60% in standard formula and 85–90% in gentle and lactose-free formulations [144]. Importantly, metabolic effects differ across formula types. Sucrose-based, lactose-free, and gentle formulations commonly contain higher proportions of added sugars, compared with standard lactose-based formulas [144]. These formulations have been most implicated in promoting increased energy intake and accelerated weight gain [145,146]. Recent proposals and guidance documents have highlighted the need to reduce added sugars in infant formulas and better match the macronutrient composition of human milk, reflecting growing recognition of potential metabolic risks [144,147,148]. However, enforceable regulations have not yet been established and practice varies across products.

The timing of complementary feeding also influences early metabolic trajectories. Introduction of solid foods before 4 months of age is associated with higher energy intake, rapid infant weight gain, and increased obesity risk in childhood [149,150,151]. Early complementary feeding may disrupt the development of satiety signaling and promote a preference for energy-dense foods [152,153,154,155]. As a result, hepatic substrate load is increased during a critical period of hepatic growth and metabolic programming [111].

Rapid weight gain represents a key intermediate phenotype linking early feeding exposures, including formula composition, sugar intake, and complementary foods, to later MASLD [143,156]. Accelerated weight gain reflects both increased caloric intake and altered appetite regulation, leading to preferential fat deposition, insulin resistance, and elevated flux of fatty acids and simple sugars to the liver [157]. Together, these processes promote de novo lipogenesis and early hepatic lipid accumulation [158,159]. Experimental sugar-reduction studies in children lower de novo lipogenesis and liver fat, supporting a causal role for dietary sugars in hepatic steatosis [160,161,162]. Studies such as these raise concerns that the high added sugar content of many commercial formulas could contribute to rapid weight gain, early adiposity, increased cardiometabolic risk, and downstream liver fat accumulation.

### 5.2. Childhood and Early Adolescent Dietary Influences

As children transition into middle childhood and early adolescence, cumulative dietary patterns, environmental exposures, and genetic susceptibility begin to interact. The EPOCH (Exploring Perinatal Outcomes Among Children) study found that increased consumption of fiber, vegetable protein, and polyunsaturated fats from childhood (~10 years) to adolescence (~16 years) was associated with lower hepatic fat, whereas greater intake of animal protein predicted higher hepatic fat [163]. Examination of gene-diet interaction revealed that the *PNPLA3* rs738409 risk variant strengthened the dietary associations with hepatic fat [163]. Specifically, the inverse associations of fiber and vegetable protein and a positive association of saturated fat were markedly stronger in carriers of the risk allele. Beyond macronutrients, fructose-rich foods and beverages exert disproportionate metabolic effects during childhood [159,164]. Fructose is taken up by the liver independent of insulin, rapidly generates triose-phosphates, and is a potent substrate for de novo lipogenesis [159,164]. Fructose-specific pathways amplify hepatic lipid accumulation and oxidative stress more strongly than glucose loads [165,166].

The evidence linking nutrient intakes to hepatic fat primarily derives from cross-sectional studies, which cannot assess causality [35,42,167,168,169,170]. Longitudinal studies are scarce. No associations were observed between energy-adjusted macronutrient intakes at different time points in childhood and hepatic fat at 17 years [171]. Likewise, no association between dietary pattern in childhood with later hepatic fat was found in the EPOCH cohort, although adherence to a healthier diet was associated with lower hepatic fat [172]. Pattern-based analyses in other cohorts indicate that Western or ultra processed diet consumption, characterized by high intake of refined carbohydrates, processed meats, and added sugars are consistently associated with higher hepatic fat [173,174,175]. In contrast, greater adherence to a Mediterranean-type diet predicts lower steatosis and improved insulin sensitivity [176,177,178].

Complementary evidence links dietary inflammatory potential with disease severity. In a cross-sectional cohort of 125 children and adolescents with MASLD, higher Dietary Inflammatory Index (DII) scores were associated with 4-fold greater odds of severe steatosis and higher FIB-4 scores [179]. Each unit increase in DII corresponded to a 2.6-fold increase in odds of more severe steatosis, suggesting that pro-inflammatory dietary patterns exacerbate hepatic injury even after MASLD onset. Physical activity, although not the focus of this review, is an important modifier of these relationships. Higher habitual activity levels attenuate hepatic fat accumulation and improve insulin sensitivity in children, partially counteracting the metabolic consequences of suboptimal diet quality [180,181].

Although randomized trials in older children demonstrate that both Mediterranean and low-fat diets reduce hepatic steatosis and improve insulin sensitivity [182,183], such studies primarily target secondary prevention. The current review aims to focus on early-life determinants—prenatal through childhood—when hepatic metabolic pathways are still being established. Dietary exposures occurring after approximately three years of age increasingly reflect environmental and behavioral influences more than developmental programming. During this developmental period, diet-driven shaping of the gut microbiome may further influence hepatic metabolism through short-chain fatty acid production, modulation of intestinal permeability, and microbial endotoxin signaling; however, detailed microbiome mechanisms are reviewed elsewhere [184,185]. Readers interested in dietary interventions in older children and adolescents are referred to recent comprehensive reviews [46,47,161].

**Table 3:** Summary of Dietary Studies in Early Childhood with Liver Fat and MASLD.

Authors	*n*	Design	Age (y)	Exposure	Main Findings
Nobili [129]	191	O	3–18	BF	↓ MASH and fibrosis
Ayonrinde [65]	1170	P	17	BF	BF ≥ 6 months → ↓ MASLD
Gale [131]	70	P	13 d 6–12 wk	BF	No D in IHCL (BF vs. FF)
Vogelezang [130]	4444	P	10	BF	No D with LFF
Geurtsen [142]	1940	O	10	SCB	↑ consumption associated with ↑ MASLD
Cohen [163]	358	L	16	D Diet (10–16 y)	↓ Fiber/Veg Pro, ↑ Animal Pro → ↑ HF

O: Observational; P: Prospective; L: Longitudinal; IHCL: Intrahepatocellular lipid; BF: Breastfeeding; FF: Formula feeding; LFF: Liver fat fraction; SCB: Sugar-containing beverage intake. ↑: increase; ↓: decrease.

## 6. Methodological Limitations

Despite evidence for a developmental continuum, the current body of literature faces significant methodological limitations. Variability in study design, dietary assessment, outcome measures, and follow-up duration complicates comparisons and limits causal inference [52]. Likewise, inconsistent use of terminology and diagnostic tools contributes to variability. Because most of the studies summarized here utilized imaging-based measures of hepatic steatosis or older NAFLD terminology, extrapolation to MASLD, as currently defined, must be made with caution. Precision is also reduced by reliance on surrogate hepatic fat measures such as fatty liver index, rather than biopsy-confirmed outcomes.

Additional limitations include small sample sizes in many pediatric cohorts, heterogeneity in age and metabolic risk, and inconsistent diagnostic criteria. Dietary intake is typically self-reported and seldom validated biochemically, contributing to exposure misclassification. Moreover, controlled feeding studies, which could establish causality, are difficult to implement for ethical and logistic reasons. Consequently, most evidence is derived from observational designs that cannot fully address residual confounding or disentangle intrauterine influences from shared postnatal environments.

Reported effect sizes are heterogenous across populations and study contexts. While many studies link maternal obesity, gestational diabetes, and diet quality to offspring hepatic outcomes, the magnitude and consistency of associations vary. For example, the impact of gestational diabetes on child hepatic fat differs by maternal BMI, ethnicity, and timing of diagnosis, with some cohorts showing strong associations and others reporting attenuated or null effects [52,128,186]. Similar variability is observed for maternal dietary patterns, gestational weight gain, and early postnatal feeding exposures. This heterogeneity underscores the need for harmonized, adequately powered longitudinal cohorts to improve the interpretation of findings.

## 7. Opportunities for Prevention and Intervention

The established reversibility of developmental programming presents a potential pathway for intervention. Preclinical studies demonstrate that optimizing maternal diet or supplementing with compounds such as resveratrol can attenuate fetal hepatic injury, supporting a life-course approach to prevention. Translating these insights into clinical practice requires early, multilevel interventions that target key windows of susceptibility.

Nutrition-focused strategies could include optimizing maternal nutrition prior to and during pregnancy, supporting exclusive breastfeeding for at least six months, limiting added sugars in infant formulas and complementary foods, and fostering nutrient-rich diets from infancy onward [187,188]. In parallel, public health and policy approaches might involve implementing community programs that promote healthy eating, providing guidance on the timing and composition of complementary feeding, establishing labeling standards for infant products, and integrating dietary counseling into routine pediatric care.

The clinical relevance of these efforts is clear: these early-life interventions align with emerging pediatric MASLD guidelines and provide actionable pathways for preventing and mitigating disease progression [189,190]. Early identification of at-risk populations, combined with supportive nutrition and lifestyle interventions, has the potential to reduce hepatic fat accumulation and improve long-term metabolic outcomes in the young.

## 8. Conclusions

Together, these findings emphasize that pediatric MASLD should not be viewed solely as a consequence of childhood lifestyle, but rather as the culmination of biological trajectories established before clinical diagnosis. Viewing MASLD through a developmental lens provides a framework for identifying vulnerable windows, refining risk stratification, and informing preventive strategies that begin prior to birth and extend through early childhood.

Future research should prioritize establishing longitudinal, harmonized cohorts with standardized dietary and hepatic outcome measures, integrating mechanistic studies to clarify nutrient-specific effects on hepatic development, and evaluating interventions across diverse populations to understand modifiers such as genetics, ethnicity, and socioeconomic status. Addressing these priorities will enhance the evidence base for effective, early-life prevention strategies and support translation into public health policy and clinical practice.

## Figures and Tables

**Table 2:** Key Risk and Protective Factors Aligned with DOHaD Model *.

Factor	Association with Pediatric MASLD Risk	Potential Role in DOHaD Programming
Maternal pre-pregnancy overweight/obesity	Consistently identified as a modifiable risk factor	Programs fetal liver for high lipid storage due to nutrient oversupply
Breastfeeding (≥6 months)	Frequently associated with duration-dependent protective effect	Promotes a slower, healthier growth trajectory and provides bioactive factors that modulate metabolism
Rapid post-natal catch-up growth	May contribute to later hepatic steatosis	Exacerbates metabolic stress on an in utero-programmed liver, accelerating fat accumulation

* As derived from the comprehensive synthesis by Querter et al. [128].

## Abbreviations

The following abbreviations are used in this manuscript:

ACC2	acetyl-CoA carboxylase 2
ALSPAC	Avon Longitudinal Study of Parents and Children
ALT	alanine aminotransferase
aOR	adjusted odds ratio
BMI	body mass index
CD36	cluster of differentiation 36
CI	confidence interval
DAMPs	damage-associated molecular patterns
DII	Dietary Inflammatory Index
EPOCH	Exploring Perinatal Outcomes Among Children
ESPRESSO	Epidemiology Strengthened by Histopathology Reports in Sweden
FABP	fatty acid binding protein
FATP	fatty acid transport protein
FATs	fatty acid transporters
FFAs	free fatty acids
FIB-4	fibrosis-4 index
GCKR	glucokinase regulatory protein
GDM	gestational diabetes mellitus
IHCL	intra-hepatocellular lipid
MAFLD	metabolic associated fatty liver disease
MASH	metabolic dysfunction-associated steatohepatitis
MASLD	metabolic dysfunction-associated steatotic liver disease
MRS	magnetic resonance spectroscopy
NAFLD	nonalcoholic fatty liver disease
NASH	nonalcoholic steatohepatitis
NLRP3	NLR family pyrin domain containing 3
OR	odds ratio
PNPLA3	patatin-like phospholipase domain-containing 3
SPCS	Shanghai Prenatal Cohort Study
SREBP-1c	sterol regulatory element binding protein 1c
TGs	triglycerides
TM6SF2	transmembrane 6 superfamily 2
TLR4	toll-like receptor 4
VLDL	very low-density lipoprotein
